# Comparative Analysis of Complete Chloroplast Genomes of Nine Species of *Litsea* (Lauraceae): Hypervariable Regions, Positive Selection, and Phylogenetic Relationships

**DOI:** 10.3390/genes13091550

**Published:** 2022-08-28

**Authors:** Weicai Song, Zimeng Chen, Wenbo Shi, Weiqi Han, Qi Feng, Chao Shi, Michael S. Engel, Shuo Wang

**Affiliations:** 1 College of Marine Science and Biological Engineering, Qingdao University of Science and Technology, Qingdao 266042, China; 2 Plant Germplasm and Genomics Center, Germplasm Bank of Wild Species in Southwest China, Kunming Institute of Botany, the Chinese Academy of Sciences, Kunming 650204, China; 3 Department of Ecology & Evolutionary Biology, University of Kansas, Lawrence, KS 66045, USA

**Keywords:** *Litsea*, chloroplast genome, structural variations, genetic relationship

## Abstract

*Litsea* is a group of evergreen trees or shrubs in the laurel family, Lauraceae. Species of the genus are widely used for a wide range of medicinal and industrial aspects. At present, most studies related to the gene resources of *Litsea* are restricted to morphological analyses or features of individual genomes, and currently available studies of select molecular markers are insufficient. In this study, we assembled and annotated the complete chloroplast genomes of nine species in *Litsea*, carried out a series of comparative analyses, and reconstructed phylogenetic relationships within the genus. The genome length ranged from 152,051 to 152,747 bp and a total of 128 genes were identified. High consistency patterns of codon bias, repeats, divergent analysis, single nucleotide polymorphisms (SNP) and insertions and deletions (InDels) were discovered across the genus. Variations in gene length and the presence of the pseudogene *ycf1^Ψ^*, resulting from IR contraction and expansion, are reported. The hyper-variable gene *rpl16* was identified for its exceptionally high Ka/Ks and Pi values, implying that those frequent mutations occurred as a result of positive selection. Phylogenetic relationships were recovered for the genus based on analyses of full chloroplast genomes and protein-coding genes. Overall, both genome sequences and potential molecular markers provided in this study enrich the available genomic resources for species of *Litsea*. Valuable genomic resources and divergent analysis are also provided for further research of the evolutionary patterns, molecular markers, and deeper phylogenetic relationships of *Litsea*.

## 1. Introduction

*Litsea* is an evergreen tree or shrub and is one of the most diverse genera (about 400 species) in the family Lauraceae (Mesangiospermae: Magnoliids: Laurales). It is widely distributed in tropical and subtropical Asia, North and South America [1,2], and 74 species are located in China, at a maximum elevation of 2700 m above sea level [3] Species of *Litsea* are utilized in a wide range of applications, covering medical, agricultural, industrial, and many other fields. *Litsea* can be used to treat a variety of conditions such as diarrhea, stomach pain, indigestion, the common cold, gastroenteritis, diabetes, edema, arthritis, asthma, pain, and trauma [1]. In addition, *Litsea* is also known for the highly effective properties of its essential oil against food-borne pathogens [4]. Its essential oils can also be resistant to several types of bacteria, have antioxidant, anti-parasitic, acute toxicity, genotoxic, and cytotoxic properties, and can even prevent several types of cancer [5,6,7]. Despite the pharmaceutical applications of *Litsea*, it is also widely used as feed for silkworm pupae, especially for muga silkworms (*Antheraea assama*) [5]. In comparison with ordinary silk produced from other food sources, muga silk produced from *Litsea* possesses a higher value and is considered to be of better quality, as reflected in its creamy and lustrous appearance and texture. Some representative species of *Litsea* are industrially important and have been utilized extensively [6]. For instance, *Litsea cubebais* is a spice shrub of considerable economic importance. The essential oil prepared from the citric acid extracted from the plant’s body is a natural spice, with a wide number of potential applications. Moreover, it is also an important raw material for the synthesis of vital compounds, such as vitamin A [7].

Chloroplasts are organelles that occur in green plants and algae, taking the responsibility for photosynthesis and other housekeeping functions. Additionally, they are essential for nitrate and sulfate assimilation as well as the synthesis of amino acids, fatty acids, chlorophyll, and carotenoids [8]. In general, chloroplast (cp) genomes have a conservative genome structure, gene content, and gene order in most monocotyledon plants [8,9]. The complete cp genome of angiosperms is usually composed of four parts: a large single-copy (LSC) region, a small single-copy (SSC) region, and two similar inverted repeat (IR) regions, with a highly conservative structure [10]. The cp genome consists of 110 to 130 genes primarily involved in photosynthesis, transcription, and translation. The contraction and expansion of IR regions and gene and intron loss events have also occurred commonly during evolution [11]. The sequences of cp genomes can provide information for genetic relationships, gene transfer, cloning, and species domestication. The cp genome of advanced plants is inherited from a single parent [12], which can be used as an effective barcode for species identification as well as the development of other potential identification markers [13]. Identification of cp genomes promotes the sustainable development of plant species, their utilization in a more rigorous scientific manner, as well as for species conservation [14,15,16].

As the rapid development and iteration of methods for obtaining and analyzing whole cp genome sequences, studies on the cp genome have shown an explosive growth [17,18]. However, in the genus *Litsea*, reports were mostly focus on chemical compositions or species-specific genomic traits [19]. Genetic resources for *Litsea* still need to be supplemented. Moreover, studies of the selection pressure and high diversity sequences within the genus *Litsea* are greatly in demand. Therefore, a detailed assembly and annotation of the complete cp genomes of various species within *Litsea* will greatly enrich the existing database, deepen the genetic recognition of the genus, and contribute to phylogenetic, evolutionary, developmental, conservation, and taxonomic investigations. Advancing our taxonomic knowledge for *Litsea* will enable us to refine conservation efforts and the utilization of natural resources, providing sufficient genetic resources for artificial breeding and drug development. In this study, we first sequenced and assembled the complete cp genomes of nine species of *Litsea*. A comparative analysis was performed, including gene features, GC content, codon usage, IR junction, repeats, Ka/Ks value, as well as nucleotide diversity (Pi). Results of analysis provide informative and valid data regarding the genotype and suitable DNA markers. Moreover, using 21 species from *Litsea*, evolutionary relationships within the genus were analyzed using the complete cp genome as well as protein-coding sequences. Ultimately, this study provides a reliable resource for further utilization and conservation of genetic resources for *Litsea*.

## 2. Materials and Methods

### 2.1. Sample Collection, DNA Extraction, and Sequencing

In this study, samples of nine species of *Litsea* were collected from the Plant Germplasm and Genomics Center, Kunming Institute of Botany, the Chinese Academy of Sciences. The process of sample collection was approved by the Kunming Institute of Botany and local policy and deposited in the Evolutionary Biology Laboratory of Qingdao University of Science and Technology. Fresh leaf tissues were collected without apparent disease symptoms and preserved in silica gel. Total genomic DNA was extracted from 150 mg of silica-dried leaf tissues using modified CTAB [20]. The quantity and quality of the extracted DNA was assessed by spectrophotometry while the integrity was evaluated using a 1% (*w*/*v*) agarose gel electrophoresis [19] The Illumina TruSeq Library Preparation Kit (Illumina, San Diego, CA, USA) was used to prepare approximately 500 bp of paired-end libraries for DNA inserts, according to the manufacturer’s protocol. These libraries were sequenced on the Illumina HiSeq 4000 platform in Novogene (Beijing, China).

### 2.2. Chloroplast Genome De Novo Assembly and Annotation

The raw data were preprocessed using Trimmomatic 0.39 software [21], including the removal of adapter sequences and other sequences introduced during sequencing, the removal of low-quality and over-N-base reads, etc. The quality of newly produced clean short reads was assessed using FASTQC v0.11.9 [22] and MULTIQC software [23]. High-quality data with Phred scores averaging above 35 were screened out. According to the reference sequence (*Litsea glutinosa*, KU382356), the chloroplast-like reads were isolated from clean reads by BLAST [24]. Short reads were *de novo* assembled into long contigs with SOAPdenovo 2.04 [25] by setting kmer values of 35, 44, 71, and 101. Finally, the long-contigs complete sequence expansion and gap filling was done by Geneious ver 8.1 [26], which forms the complete cp genome. The complete cp genome was further validated and calibrated using *de novo* splicing software NOVOplsty 4.2 [27]. GeSeq [28] was used to annotate the assembled genomes, and tRNAscanSE ver 1.21 [29] was applied to detect tRNA genes with default settings. RNAmmer [30] was used to validate rRNA genes with default settings. As a final check, we compared the results with the reference sequence and corrected misannotated genes by GB2Sequin [31] by manual selection. The circular map of the genomes was drawn using CHLOROPLOT [32]. The nine newly assembled *Litsea* cp genomes were deposited in GenBank with the accession numbers NC_056809–NC_056817.

### 2.3. Analysis of Chloroplast Genome Characteristics

Information regarding the GC content, genome length, and number of each region in cp genomes was obtained using Geneious ver 8.1 software [26]. Relative synonymous codon usage (RSCU) was calculated by the Computer Codon Usage Bias function in MEGA X [33]. SSRs were identified using MISA [34], with a setting of ten repeats for mononucleotide SSRs, four for dinucleotide and trinucleotide SSRs, and three for tetranucleotide, pentanucleotide, and hexanucleotide SSRs. REPuter [35] was used to identify four types of repeats with the minimum repetition unit set as 20 bp and the maximum as 300, and the remaining options set to default parameters.

### 2.4. Comparative Analysis

To compare the gene differences among the nine species, *Litsea garrettii* (NC_050349) was selected as the reference species and the online comparison tool mVISTA [36] was used for sequence alignment. IRscope [37] was used to detect and visualize the contraction and expansion of IRs boundaries. SNP and InDels were detected using Geneious ver 8.1. The Ka/Ks value was batch evaluated by TBtools with the NG method [38]. DnaSP 6 was used to analysis the nucleotide diversity (Pi) value [39].

### 2.5. Phylogenetic Analysis

We downloaded 12 further cp genomes of *Litsea* from NCBI (National Center for Biotechnology Information). Two species from Lauraceae but in different genera, *Actinodaphne obovate* and *Neolitsea sericea*, were selected as outgroups to root our phylogenetic networks. A total of 23 species were compared for phylogenetic evaluation using maximum likelihood (ML) and Bayesian inference (BI) approaches. MAFFT v7 was used to perform multiple genome alignment [40], and we used the complete cp genome sequence data as well as a separate dataset of 64 protein-coding genes shared by all species to construct individual maximum likelihood (ML) topologies. The GTR+G+I model was evaluated as the best suit model for both CDS and all cp sequences by applying the Bayesian information criterion (BIC) using jmodeltest v2.1.7 [41]. The ML analyses were performed using MEGA X [33], and bootstrap tests were performed with 1000 replicates with tree bisection-reconnection branch swapping. MrBayes v3.2.7 [42] was used to the BI analysis, for two million generations, sampled every 100 generations, with all other settings left at their defaults and 25% of the trees discarded as burn-in.

## 3. Results and Discussion

### 3.1. Chloroplast Genome Features of Litsea

**Figure 1:**
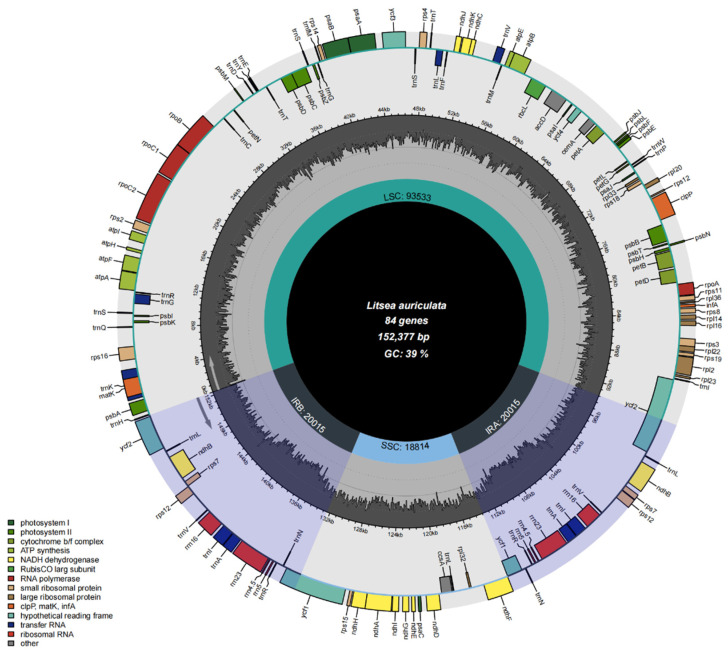
Complete genome map of the chloroplast genome for representative species *L. auriculata*. The inner gray ring is divided into four areas, clockwise, and they are SSC, IRb, LSC, and IRa. The genes in the outer ring region are transcribed clockwise, while those in the inner ring are transcribed counterclockwise. In addition, this figure also reflects the GC content; the inner ring dark gray indicates the GC content, the light gray reaction AT content. In the lower left is a legend that classifies cp genes according to their functions.

With reliable quality control, we filtered about 22.5 GB of high quality, 2 × 150 bp pair-end reads generated by the Illumina HiSeq 4000 platform. The mean coverage of sequencing was 1750 X. The cp genome features of nine species were analyzed and the total length ranged from 152,051 to 152,747 bp (Figure 1). 128 genes were found in these complete cp genomes, including 36 tRNA genes, eight rRNA genes, and 84 protein-coding genes. These genes can be divided into three categories: self-replication related, photosynthesis related, and other genes. The large subunit of ribosomal proteins, small subunit of ribosomal proteins, DNA-dependent RNA polymerase, rRNA genes, and tRNA genes belong to the Self-replication category. Photosystem I, Photosystem II, NADH oxidoreductase, Cytochrome b6/f complex, ATP synthase, and Rubisco belong to the Photosynthesis category. The remaining genes that have not been authorially classified yet were attributed to the other genes category (Table 1) [43].

Typical quadripartite and circular structures were discovered. These cp genomes contain a large single-copy (LSC) of 93,093–93,631 bp and a small single-copy region (SSC) of 18,813–18,902 bp, separated by two identical interspersed regions (IRs) of 20,014–20,117 bp. Among the four types of regions, the LSC region contained the largest number of genes, including 66 protein-coding genes and 23 tRNA genes. The SSC region contained only 11 protein-coding genes and one tRNA gene, but its average gene length was the longest at 1100 bp. Two identical IR regions contained five protein-coding genes, six tRNA genes, along with four rRNA genes (Table 1). The genome features of *Litsea* are consistent with the basic structure of cps reported by other studies [44].

We also analyzed the GC content of the complete cp genome for the nine species of *Litsea*, as well as the values of each region (Table 2). We discovered that the average GC content of the full cp genome was 39.2% for all species except for *L. sericea*, which was 39.1%. In addition, the GC content of the IR region was firmly consistent at 44.4% and significantly higher than the other two regions, which was assumed to be related to the presence of many rRNA genes [45].

### 3.2. Codon Usage Analysis

**Figure 2:**
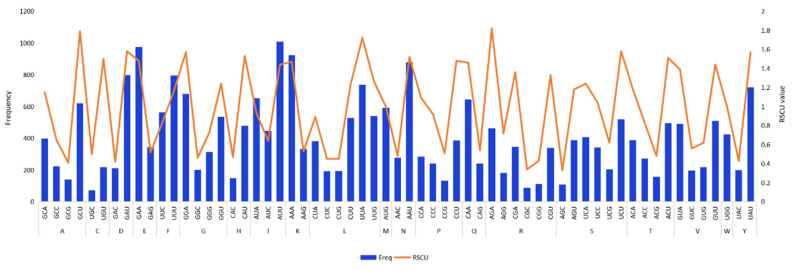
The frequency (Freq) and preference of codon usage (RSCU) in *L. moupinensis* protein-coding region. Axis of abscissae indicate each amino acid and its abbreviation as well as the respective codon, the blue bar in the figure is the frequency of codon usage, and the orange line represents codon preference.

All organisms share the same common codon table, reflecting the shared ancestry of all life. But through the process of biological evolution, disproportionate biases have evolved. Different species exhibit certain preferences for not only different synonymous codons, but also different proteins within the same species may show a preference for the same amino acid, a phenomenon called *codon bias*. A measurement called RSCU is commonly used to reflect the codon bias, which removes the effect of the amino acid composition of a codon [44]. Since *L. moupinensis* had the largest cp genome, we used it as an example to calculate the codon usage bias and RSCU values of 84 CDS genes. The protein-coding genes in the complete cp genome of *Litsea* consist of 84 genes coded by 61 codons, which encode 20 amino acids. The results showed that Leu (UUA), Ala (GCU), and Arg (AGA) are the most frequently used amino acids, while Ser (AGC) and Arg (CGC) are the least abundant amino acids (Figure 2). RSCU values greater than one mean that there is significant codon bias. This results in a different use of amino acids, which correlates with protein-positive bias [45]. Analysis of RSCU values of the codons encoding each amino acid revealed that most codons with RSCU > 1 contained either an A- or G-terminal. By contrast, RSCU values for codons that ended with a C-terminal, such as CGC (Arg), UGC (Cys), CAC (His), and AGC (Ser), were relatively low. This result was consistent with previous related reports [46].

### 3.3. Long Repeat and SSR Analysis

Long repeat sequences and SSRs analysis commonly exist throughout the cp genome, consisting of one to six nucleotide repeats [44]. Due to its variability at the intraspecific level, SSRs are commonly used as markers in population genetic analyses [47,48]. In the cp genome of nine species, the total number of repeats ranged from 109 (*L. chuni*) to 119 (*L. auriculata*) (Table S1). A total of 111 SSRs were detected from the cp genome of the representative species *L. moupinensis*, including 62 mononucleotide, 36 dinucleotide, tree trinucleotide, eight tetranucleotide, one pentanucleotide, and one hexanucleotide repeats. In general, the SSR number decreased along with the increase in nucleotide number. The percentage of tri-, tetra-, penta-, and hexa-nucleotide repeat sequences detected were remarkably lower than that of mono- and di-nucleotide repeat. Mono-nucleotide repeats were the largest class of SSRs that consisting of 56.97% of all repeats. These repeats were notably rich in A/T bases, causing the differences in terms of base content, which was in line with other angiosperm species [49]. We also analyzed the distribution of SSRs in LSC/SSC/IR regions. The number of SSR markers in the LSC region of nine species of *Litsea* ranged from 79 to 87, far exceeding that of SSC (19) and IR regions (12). In particular, IR region contains the lowest number of SSRs, which further demonstrates the high degree of conservatism of IR regions. This correlated phenomenon was previously reported in other angiosperms studies [50].

**Figure 3:**
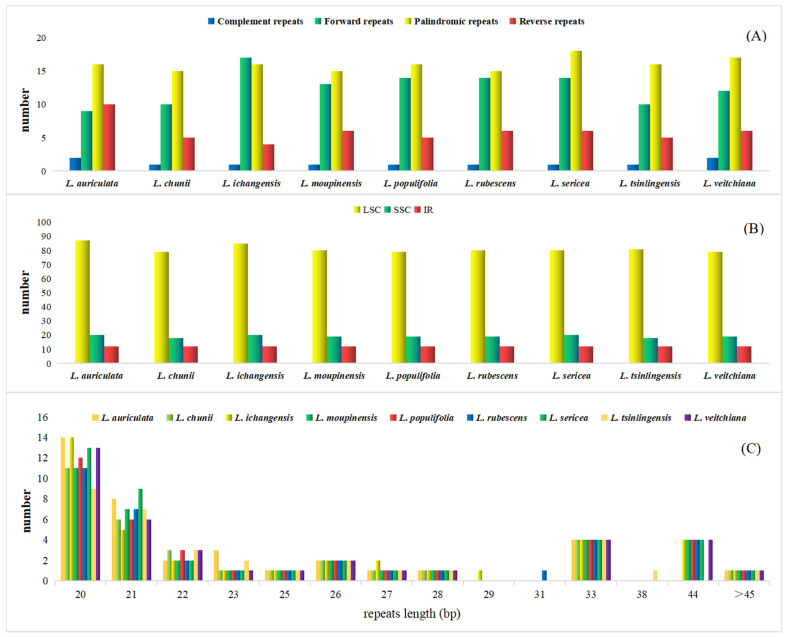
Comparison of microsatellites and oligonucleotide repeats in the chloroplast genomes of *Litsea* species. (**A**) The number of SSR markers in the LSC/SSC/IR region for nine *Litsea* species. (**B**) Number of four long repeat sequences in nine species: complement repeats. F represents forward repeats, P represents palindromic repeats, R represents reverse repeats, C represents complement repeats. (**C**) number of long repeat sequences with different lengths in nine species. Different colors in the figure represent different long repeat sequence types. Species from left to right are: *L. auriculata*, *L. chunii*, *L. ichangensis*, *L. moupinensis*, *L. populifolia*, *L. rubescens*, *L. sericea*, *L. tsinlingensis*, *L. veitchiana*.

Some repeats larger than 30 bp in length are called long repeat sequences, which increase the rearrangement of the cp genome [51]. We investigated the interspersed repeated sequences (IRs) including four types of long repeat sequences: complement repeats (C), forward repeats (F), palindromic repeats (P), reverse repeats (R). In general, palindromic repeatswere richest in most species, followed by forward repeats and reverse repeats. Complement repeats (C) were notably rare among all species. However, in the cp genome of *L. ichangensis*, the number of forward repeats were slightly higher than that of palindromic repeats (16). What more, in the cp genome of *L. auriculata*, the ratio of reverse repeats (R) was more than that of forward repeats (F), which is also different from the other eight species (Figure 3A). Most repeats were found in LSC region, leaving SSC and R regions far behind. This pattern is highly consistent in nine *Litsea* species analyzed (Figure 3B). We also measured the number of long repeat sequences with different lengths (Figure 3C). It was found that long repetitive sequences of length of 20 and 21 bp were most common, while the remainder decreased in number with an increase in length in general. The repeats with 29, 31, and 38 bp in length were almost absent. However, in 33 bp and 44 bp, the repeats number presented to be tied for third place suddenly. This phenomenon varied from different species, which may be affected by unknown molecular mechanisms [52,53].

### 3.4. IR Contraction Analysis and Sequence Identity Plot

**Figure 4:**
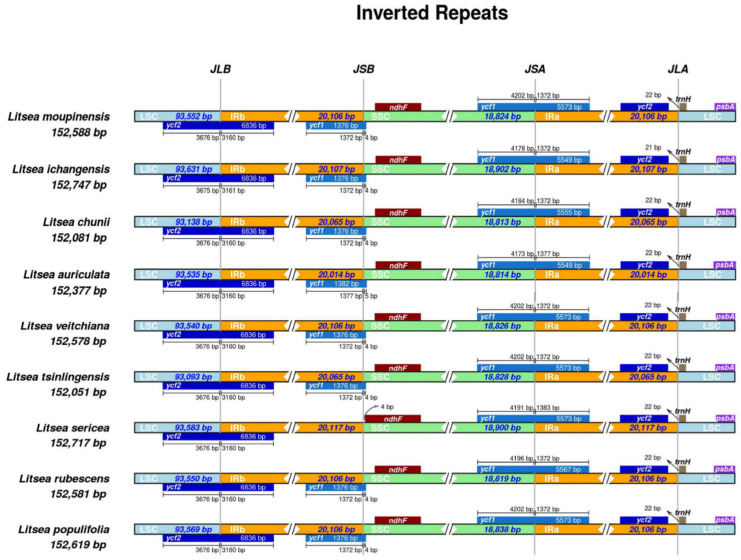
Comparison of SSC, LSC, IRa, and IRb boundary regions in the chloroplast genomes of nine species of *Litsea*.

The contraction and expansion of IR regions contribute greatly to variations of cp genomes among different species, resulting in gene duplication, deletion, and the generation of pseudogenes. Studying the characteristic genes of the border region contributes to species identification and phylogenetic analyses [54]. In this study, we analyzed and visualized the genes located in the junction region of LSC and IRa (JSa) as well as the junction of SSC and IRb (JSb) in the cp genome of the nine species of *Litsea* (Figure 4). JLa represents the junction between LSC and IRa, and the same applies for JLb. In this study, we observed that genes located in the junction of four regions were highly conserved, with only a few variations. Most genes located at cp genome junctions in all nine species differed only in the distance to their corresponding boundaries, such as *ycf2*, *ndhF*, *trnH*, and *psbA*. To be more specific, the *ycf2* gene spans LSC/IRb and is distributed in both regions of similar length, with the LSC region being slightly longer. The *ndhF* gene exists among nine species, completely located in SSC and a short distance from IRb except for *L. sericea*, of which theirs was longer and closer to the JSb boundary. The *trnH* gene is located in the LSC region, adjacent to the IRa/LSC border, and is 21–22 bp in length. *PsbA* is located entirely in the LSC region. Yet, notable variations were found. The *ycf1* gene was absent in this junction, while the remaining eight species contain *ycf1^Ψ^* (pseudocopy, 5′ end missing) in JSb, which spans JSb with only 4–5 bp of length, located in SSC. Apart from that, the contraction and expansion event located in the JSb was greater than that of the JLa boundary. This pattern is consistent with previous IR region research [55].

**Figure 5:**
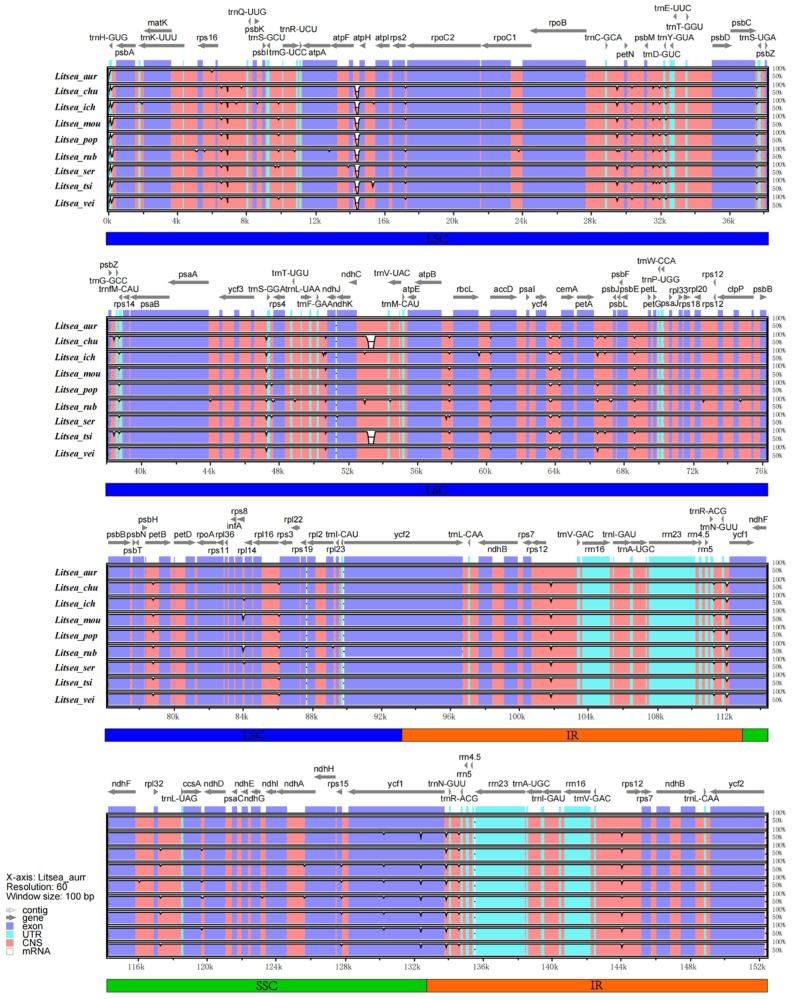
Identification map of chloroplast genome of nine species of *Litsea*. From top to bottom: *L. auriculata*, *L. chunii*, *L. ichangensis*, *L. moupinensis*, *L. populifolia*, *L. rubescens*, *L. sericea*, *L. tsinlingensis*, *L. veitchiana*. The gray arrows above indicate the extension direction of the gene, and purple indicates the exon, blue indicates the untranslated region, pink indicates the non-coding sequences, and the grayish part indicates mRNA.

The whole sequence identity plot of nine species within *Litsea* was analyzed using mVISTA with *L. garretti* (NC_050349) set as the reference sequence for comparison (Figure 5). Genome sequences of the nine *Litsea* exhibited a high degree of concordance. In this study, we revealed that most of the variations in the cp genome of different species were distributed in CNS (non-coding sequences) regions. Notable high-divergent regions in CNS were *atpF*–*atpH* and *ndhC*–*trnV-UAC*, the divergent value of which exceeded 100%. Other variant regions include: *rps16*–*trnQ-UUG*, *ycf4*–*cemA*, *rps8*–*rpl14*, and *rps12*–*trnV-GAC*. Some of the coding genes, such as *ndhK*, *ndhF*, and *ycf1*, were found to were contain variable regions. In general, the divergence in the IR region was significantly smaller than that in the LSC and SSC regions, a result comparable to the previous divergence analysis [50].

### 3.5. SNP and InDels

To further explore the divergence of nucleotides, we compared and analyzed single nucleotide polymorphisms (SNPs) and insertions and deletions (InDels) using *L. garrettii* (NC_050349) as a reference sequence. The polymorphism ratio of transition substitution (Ts) was higher than transversion substitutions (Tv) in the LSC region of nine cp genomes (Table 3). The most substitutions were located in the LSC region, while IR regions contained the lowest rate of polymorphisms. This result is consistent with previous studies [56]. In terms of transition substitutions, the polymorphism ratios of A/G and C/T were almost the same, although the former took up a slightly larger proportion, with only three exceptions (*L. auriculata*, *L. chunii*, and *L. tsinlingensis*). As for transversion substitutions, the polymorphism ratios of A/T and C/G were greatly lower than that of A/C and G/C substitutions. The same pattern applied for InDels (Table 4). LSC presented the largest number of InDels in comparison with IR and SSC regions, while the average length of InDels in IR regions was the longest, with the longest variation length being 678 bp (*L. tsinlingensis*).

In particular, we found that in the cp genome of *L. auriculata*, the average length of InDels located in the IR regions contained a considerable number of small InDels rather than only several long InDels, as was found in the other eight species, causing its average length to be three times shorter than others. This result indicated that *L. auriculata* may have experienced some degree of mutation during its evolution that differed from its related species (*Vaccinium*) [57].

### 3.6. Nucleotide Divergence and Selection Pressure

**Figure 6:**
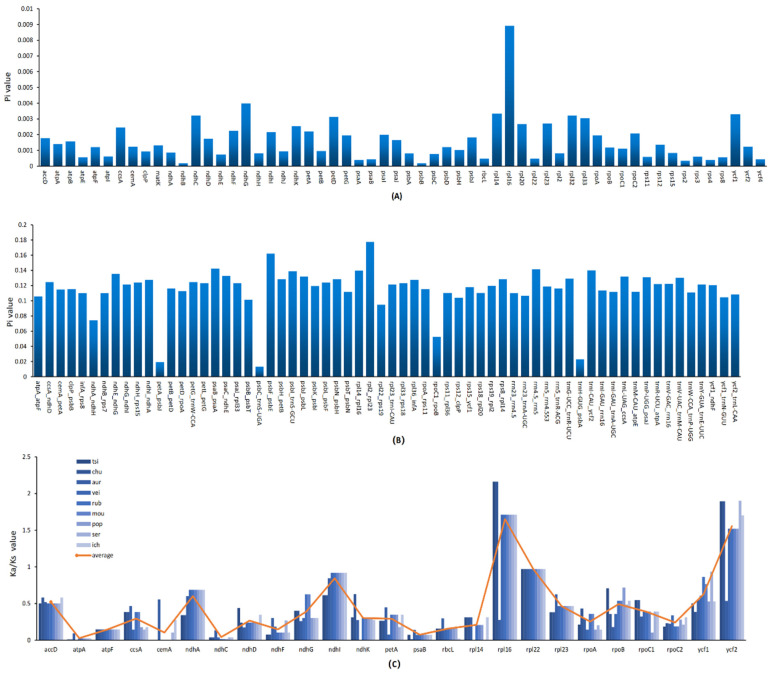
Nucleotide diversity in chloroplast genomes of nine species of *Litsea*. The abscissa represents the position, and the red line represents the average of the nucleotide variations of the nine species. (**A**) Pi values for each gene region. (**B**). Pi values for each intergenic region. (**C**) Ka/KS values for each gene.

Despite general consistency, variations occurred frequently during the evolutionary process, forming different genotypes and phenotypes. These nucleotide variations (Pi) could be distinguished as high divergent regions [58]. Some may accumulate through generations to better adapt to the environmental changes, which is called positive selection. In bioinformatics, the Ka/Ks value is commonly used to evaluate selection pressure. Here, we calculated and analyzed the Pi value of 79 unique protein-coding genes, 101 IGS (intergenic spacer) sequences, and the Ka/Ks value of 79 unique protein-coding genes (Table S2). Most of the protein-coding genes possessed relatively low diversity, while the *rpl16* gene presented with an extremely high Pi value (0.00892) among all samples (Figure 6A). However, in IGS regions, the Pi values of 64 genes out of 101 exceeded 0.01 (Figure 6B). Moreover, 54 among them surpassed 0.1. As for selection pressure, after filtering genes with no value, the Ka/Ks value of 23 of 25 genes were less than one using *L. Garrettii* (NC_050349) as a reference sequence. In other words, these genes were under negative selection pressure. Only two genes, *rpl16* and *ycf2*, presented with a Ka/Ks value of greater than one, undergoing positive selection. No gene presented with a suggested neutral selection (Figure 6C).

The hyper-variable regions detected in this study may provide a potential molecular marker for further studies. In particular, the *rpl16* gene possesses both a high Pi value and Ka/Ks value at the same time. This might suggest that the *rpl16* gene went through a great mutation that was crucial to the evolution process of *Litsea* species. Although studies have reported *rpl16* to be one of the highly divergent genes [59] and a positive selection site [60], as unique and significant as the present study is this is not a common in studies of other angiosperms. The other positive selection site, the *ycf2* gene, was more commonly described in previous studies [61,62].

### 3.7. Phylogenetic Analysis

**Figure 7:**
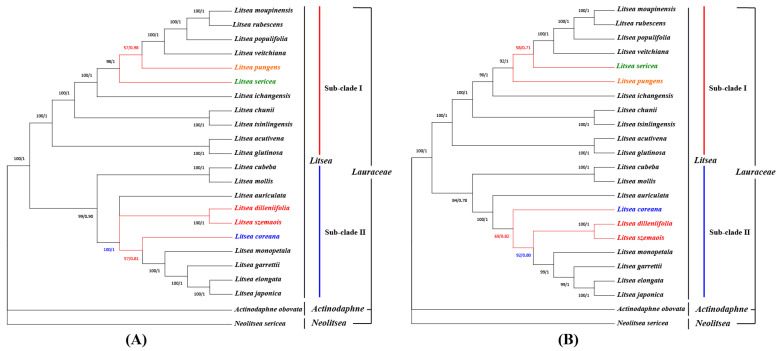
Phylogenetic analysis. (**A**) Phylogenetic tree based on the complete chloroplast genome. (**B**) Phylogenetic tree based on 64 sets of protein-coding genes. *N. sericea* and *Actinodaphne obovata* served as out groups. The colored branches show the difference between two trees. Numbers at branch nodes are bootstrap values and posterior probability.

The expanding cp genome database provides an important basis for determining evolutionary relationships [56,63,64,65] Phylogenetic trees based on different data had slightly varied topologies, with trees based on the whole cp genome and CDS data having the same topology, and being more credible than trees based on the IR area and introns [61,66,67,68,69]. We found two similar topological structures with few changes based on the full cp genome and the protein-coding sequences of 23 selected species, with *N. sericea* and *A. obovate* as outgroup species (Table S3, Figure 7).

In general, the entire phylogenetic tree was divided into three main branches, with the two outgroup species representing two distinct branches, each with high bootstrap values. The first subclade consists of 11 species: *L. moupinensis*, *L. rubescens*, *L. populifolia*, *L. veitchiana*, *L. pungens*, *L. sericea*, *L. ichangensis*, *L. chunii*, *L. tsinlingensis*, *L. acutivena* and *L. glutivena*. Among them, the clade of *L. chunii* and *L. tsinlingensis* and the clade of *L. acutivena* and *L. glutinosa* form sister pairs, respectively. Notably, *L. pungens* switched phylogenetic positions with *L. sericea*, with relatively low bootstrap values in both trees. Another clade included 10 species: *L. cubeba*, *L. mollis*, *L. dilleniifolia*, *L. szemaois*, *L. auriculata*, *L. coreana*, *L. monpinensis*, *L. garrettii*, *L. elongata*, and *L. japonica*. Among them, *L. cubeba* and *L. mollis* were grouped as sisters and clustered with eight other species. It is worth noting that in topology based on the complete cp genome, *L. coreana* and *L. monopetala* were sisters with low support (only 57). However, in the CDS-based tree, *L. dilleniifolia* and *L. szemaois* split into a clade that aggregated with the remaining four species (*L. monpinensis*, *L. garrettii*, *L. elongata*, and *L. japonica*), and merged with *L. coreana* to converge as a single branch. In other words, in the two different analyses, the clade consisting of *L. dilleniifolia* and *L. szemaois* switched its position with *L. coreana*. The phylogenetic trees resulting from Bayesian inference analyses (File S1) were generally consistent with the results of the maximum likelihood tree. However, in the Bayesian inference tree, the positions of *L. pungens* and *L. sericea* were consistent with the results of the maximum likelihood tree for the complete cp genome, while the relationships of *L. coreana*, *L. dilleniifolia*, and *L. szemaois* were consistent with the results of the maximum likelihood tree constructed by CDS.

The development of low-copy nuclear DNA regions to investigate phylogenetic relationships among plant taxa has attracted growing interest [70]. Therefore, many studies have tried to study the phylogenetic relationships of the genus *Litsea* using different methods, such as combined *matK* and *ITS* [71], *rpb2* [72] gene fragments, and morphological characters [73]. These studies focused on the analysis of the relationships between different genera in Lauraceae. In comparative terms, the phylogenetic relationships constructed from the complete chloroplast genome are more accurate than those constructed from a few fragments [74]. Zhang et al. (2021) [75] suggested that *Litsea* could be divided into four sub-clades through the chloroplast genome. However, our study has suggested that *Litsea* is more appropriately divided into two sub-clades (Figure 7). We discovered that both the ML tree and the BI tree had greater support values for the phylogeny reconstructed from complete cp genomes. Such different trees could originate from substitutions in the intergenic spacer regions, which illustrates the importance of non-coding regions in phylogenetic analyses [76]. Therefore, complete cp genomes can be used as a ‘super barcode’ [77], and they have been demonstrated to be effective for preventing some identification errors and the discovery of new species [78]. Despite minor differences, the phylogenetic relationships of most species in the topologies were consistent, showing similar genetic affinities in the topology, and which aligned nicely with the elevational distribution of the species [71,79].

## 4. Conclusions

In this study, we sequenced and reported the complete cp genome sequences of nine species in *Litsea*, revealing typical quadripartite circular structures. We observed the contraction and expansion of IR boundaries. This event caused gene loss, changes in gene length, and the occurrence of pseudogenes, resulting in the differences between species. In terms of alignment consistency, the LSC region had the largest number of nucleotide variants, and IR regions showed a high degree of conservation. We found that the *rpl16* and *ycf2* genes underwent great positive selection pressure. Moreover, *rpl16* gene also was found to be the only hyper-variable protein-coding gene in the gene divergent analysis, which was evaluated by the Pi value. This phenomenon is rare and further studies to unfold the molecular mechanism behind is needed.

Phylogenetic relationships within the genus were explored using two sets of data from the complete cp genome and another from 64 sets of protein-coding genes shared by 21 *Litsea* and two outgroup species. Essentially the same conclusions were obtained: *L. moupinensis* and *Litsea rubescena*, *L. chunii*, and *L. tsinlingensis* were sisters in the phylogenies and showed similar genetic relationships consistent with their elevational distributions. This study provides aid to taxonomic studies for *Litsea*, providing specific genetic markers for taxon identification and for inferring evolutionary relationships among the species. These data may also contribute to future conservation efforts as well as the practical use of these species.

## Figures and Tables

**Table 1:** Gene content of the *L. moupinensis* chloroplast genome.

Group of Genes	Gene Names	Amount
Pholosystem I	*psaA*, *psaB*, *psaC*, *psal*, *psaJ*	5
Photosystem II	*psbA*, *psbK*, *psbl*, *psbM*, *psbD*, *psbC*, *psbZ*, *psbG*, *psbL*, *psbF*, *psbE*, *psbB*, *psbT*, *psbN*, *psbH*	15
Cytochrome	*petA*, *petG*, *petL*, *petN*, *petB*, *petD*	6
ATP syntliase	*atpA*, *atpF*, *atpH*, *atpI*, *atpE*, *atpB*	6
NADH dehydrogenase	*ndhJ*, ndhB *, *ndhK*, *ndhC*, *ndhD*, *ndhF*, *ndhE*, *ndhG*, *ndhl*, *ndhA*, *ndhH*	12
RubisCO large subunit	*rbcL*	1
RNA polymerase	*RpoCl*, *rpoC2*, *rpoB*, *rpoA*	4
Ribosomal proteins (SSU)	*rps16*, rpsl2 *, *rps2*, *rps14*, *rps4*, *rps18*, rps7 *, *rps11*, *rps8*, *rps3*, *rps19*, *rps15*	14
Ribosomal proteins (LSU)	*rpl33*, *rpl20*, *rpl36*, *rpll4*, *rpll6*, *rpl22*, *rpl2*, *rpl23*, *rpl32*	9
Transfer RNAs	*trnH-GUG*, *trnK-UUU*, *trnQ-UUG*, *trnS-GCU*, *trnG-UCC*, *trnR-UCU*, *trnC-GCA*, *trnD-GUC*, *trnY-GUA*, *trnE-UUC*, *trnT-GGU*, *trnS-UGA*, *trnG-UCC*, *trnT-GGU*, *trnS-UGA*, *trnG-UCQ*, *trnM-CAU*, *trnS-GGA*, *trnT-UGU*, *trnL-UAA*, *trnF-GUU*, *trnV-UAC*, *trnM-CAU*, *trnW-CCA*, *trnP-UGG*, *trnl-CAU*, *trnA-UGC*, *trnR-ACG*, *trnL-UAG*, *trnN-GUU*, *trnR-GUG*, *trnA-UGC*, *trnl-GAU*, *trnL-CAA*	34
Ribosomal RNAs	rrn4.5 *, rrn5 *, rrn16 *, rrn23 *	8
Hypothetical chloroplast reading frames (ycf)	*ycfl*, *ycf2*, *ycf3*, *ycf4*	4
Other genes	*accD*, *clpP*, *ccsA*, *cemA*, *infA*, *rpoA*, *matK*	7

* Gene with two copies.

**Table 2:** Chloroplast genome features of nine species of *Litsea*.

*L. auriculata*	*L. chunii*	*L. ichangensis*	*L. moupinensis*	*L. populifolia*	*L. rubescens*	*L. sericea*	*L. tsinlingensis*	*L. veitchiana*
152,377	152,081	152,747	152,588	152,619	152,581	152,717	152,051	152,578
93,535	93,138	93,631	93,552	93,569	93,550	93,583	93,093	93,540
18,814	18,813	18,902	18,824	18,838	18,819	18,900	18,828	18,826
20,014	20,065	20,107	20,106	20,106	20,106	20,117	20,065	20,106
39.2	39.2	39.2	39.2	39.2	39.2	39.1	39.2	39.2
37.9	37.9	38.0	37.9	38.0	37.9	37.9	37.9	38.0
33.9	33.9	33.9	33.9	33.9	33.9	33.9	34.0	34.0
44.4	44.4	44.4	44.4	44.4	44.4	44.4	44.4	44.4
128 (113)	128 (113)	128 (113)	128 (113)	128 (113)	128 (113)	128 (113)	128 (113)	128 (113)
84 (79)	84 (79)	84 (79)	84 (79)	84 (79)	84 (79)	84 (79)	84 (79)	84 (79)
9 (4)	8 (4)	8 (4)	8 (4)	8 (4)	8 (4)	8 (4)	8 (4)	8 (4)
36 (30)	36 (30)	36 (30)	36 (30)	36 (30)	36 (30)	36 (30)	36 (30)	36 (30)
NC_056809	NC_056810	NC_056811	NC_056812	NC_056813	NC_056814	NC_056815	NC_056816	NC_056817
49.12	49.35	49.06	49.12	49.11	49.12	49.15	49.37	49.12
8.51	7.54	8.81	8.82	8.82	8.95	8.81	8.85	8.82
30.19	29.92	30.93	30.85	30.86	1.36	30.83	29.90	30.85
13	13	13	13	13	13	13	13	13

**Table 3:** The number of SNP types in LSC, IR and SSC regions of nine *Litsea* chloroplast genomes.

Species	Region	Transition Substitutions		Transversion Substitutions			
		A/G	C/T	A/T	A/C	C/G	G/T
*L. auriculata*		109	106	25	46	6	63
*L. chunii*	Large	137	129	20	52	11	80
*L. ichangensis*		129	139	26	58	11	75
*L. moupinensis*	single	134	139	21	58	10	78
*L. populifolia*	copy	129	138	22	60	10	80
*L. rubescens*		134	140	22	58	10	78
*L. sericea*		123	129	23	55	10	78
*L. tsinlingensis*		136	127	19	56	11	73
*L. veitchiana*		127	128	21	59	10	75
*L. auriculata*		3	5	0	2	2	11
*L. chunii*	Inverted repeat	4	8	2	12	1	15
*L. ichangensis*		4	5	1	3	1	3
*L. moupinensis*		3	8	2	12	1	12
*L. populifolia*		3	6	2	12	1	11
*L. rubescens*		3	8	2	12	1	11
*L. sericea*		3	6	3	12	1	12
*L. tsinlingensis*		2	9	2	12	1	14
*L. veitchiana*		3	7	2	12	1	12
*L. auriculata*		43	47	5	21	3	16
*L. chunii*	Small	42	37	10	19	4	17
*L. ichangensis*		42	45	5	24	5	21
*L. moupinensis*		38	41	5	24	6	21
*L. populifolia*	single	37	43	4	19	5	21
*L. rubescens*	copy	38	41	5	10	6	21
*L. sericea*		38	39	6	19	5	24
*L. tsinlingensis*		42	37	5	20	4	18
*L. veitchiana*		37	43	4	18	5	22

**Table 4:** Comparative analyses of the number and average length of InDel sites in LSC, SSC, and IR regions in the complete cp genomes of nine species of Litsea.

Comparative Analyses of InDel Sites						
Species	Large Single Copy		Inverted Repeat		Small Single Copy	
	No′s of InDels	InDels′ Average Length (bp)	No′s of InDels	InDels′ Average Length (bp)	No′s of InDels	InDels′ Average Length (bp)
*L. auriculata*	86	4.40	16	103.3	18	1.3
*L. chunii*	89	8.4	3	458.7	20	1.4
*L. ichangensis*	99	3.7	5	276.0	19	1.6
*L. moupinensis*	88	3.7	4	339.0	16	1.9
*L. populifolia*	86	3.8	4	339.0	17	1.5
*L. rubescens*	88	3.9	5	272.4	16	1.9
*L. sericea*	86	3.7	4	339.0	19	2.0
*L. tsinlingensis*	87	9.1	2	678.0	19	1.4
*L. veitchiana*	88	3.7	4	339.0	18	1.8
Average	88.6	4.90	5.2	349.4	18.0	1.6

## Data Availability

The data that support the findings of this study are openly available in the Genbank database at https://www.ncbi.nlm.nih.gov/, under accession number [NC_056809 – NC_056817] (accessed on 1 September 2021).

## Supplementary Materials

The following supporting information can be downloaded at: https://www.mdpi.com/article/10.3390/genes13091550/s1, Table S1: Raw data of single sequence repeats of nine *Litsea* species, grouped by mono-, di-, tri-, tetra, penta-, and hexanucleotide repeats, along with the percentage of each group. Table S2: Raw data of Ka/Ks value and pi value of 79 protein-coding genes. Raw data of pi value of 101 IGS sequences. Table S3: Details of taxonomy and accession numbers of species mentioned in this study. File S1: The phylogenetic trees resulting from Bayesian inference analyis.

## Abbreviations

AGE, agarose gel electrophoresis; BI, Bayesian inference; CDS, Coding sequences; Cp, chloroplast; CTAB, Cetyltrimethylammonium bromid; DOGMA, Dual Organellar Genome Annotator; IGS, Intergenic spacer; InDels, insertions/deletions; IR, inverted repeat; Ka, Nonsynonymous; Ks, Synonymous; LSC, large single-copy; ML, Maximum likelihood; NJ, neighbor joining; PCGs, Protein-coding genes; Pi, Nucleotide variance; RSCU, relative synonymous codon usage; SBS, Sequencing By Synthesis; SNP, single nucleotide polymorphisms; SSC, small single-copy; SSR, simple sequence repeat.

## References

[1] Wang Y.S., Wen Z.Q., Li B.T., Zhang H.B., Yang J.H. (2016). Ethnobotany, phytochemistry, and pharmacology of the genus *Litsea*: An update. J. Ethnopharmacol..

[2] Kong D.G., Zhao Y., Li G.H., Chen B.J., Wang X.N., Zhou H.L., Lou H.X., Ren D.M., Shen T. (2015). The genus *Litsea* in traditional Chinese medicine: An ethnomedical, phytochemical and pharmacological Review. J. Ethnopharmacol..

[3] Chanderbali A.S., Werff H., Renner S.S. (2001). Phylogeny and historical biogeography of Lauraceae: Evidence from the chloroplast and nuclear genomes. Ann. Mo. Bot. Gard..

[4] Tyagi A.K., Malik A. (2011). Antimicrobial potential and chemical composition of eucalyptus globulus oil in liquid and vapour phase against food spoilage microorganisms. Food Chem..

[5] Choudhury S., Ahmed R., Barthel A., Leclercq P.A., Leclercq P.A. (1998). Composition of the stem, flower and fruit oils of *Litsea* cubeba pers. from two locations of Assam, India. J. Essent. Oil Res..

[6] Kajaria D.K., Gangwar M., Kumar D., Sharma A.K., Tilak R., Nath G., Tripathi Y.B., Tripathi J.S., Tiwari S.K. (2012). Evaluation of antimicrobial activity and bronchodialator effect of a polyherbal Drug-Shrishadi. Asian Pac. J. Trop. Biomed..

[7] Kamle M., Mahato D.K., Lee K.E., Bajpai V.K., Gajurel P.R., Gu K.S., Kumar P. (2019). Ethnopharmacological properties and medicinal uses of *Litsea cubeba*. Plants.

[8] Deng Y., Luo Y., He Y., Qin X., Li C., Deng X. (2020). Complete chloroplast genome of *Michelia shiluensis* and a comparative analysis with four Magnoliaceae species. Forests.

[9] Yang Z., Zhao T., Ma Q., Liang L., Wang G. (2018). Comparative genomics and phylogenetic analysis revealed the chloroplast genome variation and interspecific relationships of *Corylus* (Betulaceae) species. Front. Plant Sci..

[10] Song W., Chen Z., He L., Feng Q., Zhang H., Du G., Shi C., Wang S. (2022). Comparative chloroplast genome analysis of wax gourd (*Benincasa hispida*) with three Benincaseae species, revealing evolutionary dynamic patterns and phylogenetic implications. Genes.

[11] Krause K. (2011). Piecing together the puzzle of parasitic plant plastome evolution. Planta.

[12] Daniell H., Lin C.S., Yu M., Chang W.J. (2016). Chloroplast genomes: Diversity, evolution, and applications in genetic engineering. Genome Biol..

[13] Vu H.T., Tran N., Nguyen T.D., Vu Q.L., Bui M.H., Le M.T., Le L. (2020). Complete chloroplast genome of *Paphiopedilum delenatii* and phylogenetic relationships among Orchidaceae. Plants.

[14] Guo S., Guo L., Zhao W., Xu J., Li Y., Zhang X., Shen X., Wu M., Hou X. (2018). Complete chloroplast genome sequence and phylogenetic analysis of *Paeonia ostii*. Molecules.

[15] Niu Z., Xue Q., Wang H., Xie X., Zhu S., Liu W., Ding X. (2017). Mutational biases and GC-biased gene conversion affect GC content in the plastomes of *Dendrobium* genus. Int. J. Mol. Sci..

[16] Tian N., Han L., Chen C., Wang Z. (2018). The Complete Chloroplast genome sequence of *Epipremnum aureum* and its comparative analysis among eight Araceae species. PLoS ONE.

[17] Jansen R.K., Raubeson L.A., Boore J.L., DePamphilis C.W., Chumley T.W., Haberle R.C., Wyman S.K., Alverson A.J., Peery R., Herman S.J. (2005). Methods for obtaining and analyzing whole chloroplast genome sequences. Methods Enzymol..

[18] Wang Y., Wang S., Liu Y., Yuan Q., Sun J., Guo L. (2021). Chloroplast genome variation and phylogenetic relationships of Atractylodes species. BMC Genom..

[19] Ju J., Xie Y., Yu H., Guo Y., Cheng Y., Zhang R., Yao W. (2020). Major components in Lilac and *Litsea cubeba* essential oils kill *Penicillium roqueforti* through mitochondrial apoptosis pathway. Ind. Crops Prod..

[20] Liu X., Xu X., Zhao J. (2011). A new generalized P-Value approach for testing equality of coefficients of variation in k normal Populations. J. Stat. Comput. Simul..

[21] Bolger A.M., Lohse M., Usadel B. (2014). Trimmomatic: A flexible trimmer for Illumina sequence data. Bioinformatics.

[22] Babraham Bioinformatics—FastQC A Quality Control Tool for High throughput Sequence Data. https://www.bioinformatics.babraham.ac.uk/projects/fastqc/.

[23] Ewels P., Magnusson M., Lundin S., Käller M. (2016). MultiQC: Summarize analysis results for multiple tools and samples in a single report. Bioinformatics.

[24] Altschul S.F., Gish W., Miller W., Myers E.W., Lipman D.J. (1990). Basic local alignment search tool. J. Mol. Biol..

[25] Luo R., Liu B., Xie Y., Li Z., Huang W., Yuan J., He G., Chen Y., Pan Q., Liu Y. (2012). SOAPdenovo2: An empirically improved memory-efficient short-read *de Novo* assembler. Gigascience.

[26] Kearse M., Moir R., Wilson A., Stones-Havas S., Cheung M., Sturrock S., Buxton S., Cooper A., Markowitz S., Duran C. (2012). Geneious basic: An integrated and extendable desktop software platform for the organization and analysis of sequence data. Bioinformatics.

[27] Dierckxsens N., Mardulyn P., Smits G. (2017). NOVOPlasty: *De Novo* assembly of organelle genomes from whole genome data. Nucleic Acids Res..

[28] Tillich M., Lehwark P., Pellizzer T., Ulbricht-Jones E.S., Fischer A., Bock R., Greiner S. (2017). GeSeq—Versatile and accurate annotation of organelle genomes. Nucleic Acids Res..

[29] Lowe T.M., Eddy S.R. (1997). TRNAscan-SE: A program for improved detection of transfer RNA genes in genomic sequence. Nucleic Acids Res..

[30] Lagesen K., Hallin P., Rødland E.A., Stærfeldt H.H., Rognes T., Ussery D.W. (2007). RNAmmer: Consistent and rapid annotation of ribosomal RNA genes. Nucleic Acids Res..

[31] Lehwark P., Greiner S. (2019). GB2sequin—A file converter preparing custom GenBank files for database submission. Genomics.

[32] Zheng S., Poczai P., Hyvönen J., Tang J., Amiryousefi A. (2020). Chloroplot: An online program for the versatile plotting of organelle genomes. Front. Genet..

[33] Kumar S., Stecher G., Li M., Knyaz C., Tamura K. (2018). MEGA X: Molecular evolutionary genetics analysis across computing platforms. Mol. Biol. Evol..

[34] Beier S., Thiel T., Münch T., Scholz U., Mascher M. (2017). MISA-Web: A web server for microsatellite prediction. Bioinformatics.

[35] Kurtz S., Choudhuri J.V., Ohlebusch E., Schleiermacher C., Stoye J., Giegerich R. (2001). REPuter: The manifold applications of repeat analysis on a genomic scale. Nucleic Acids Res..

[36] Frazer K.A., Pachter L., Poliakov A., Rubin E.M., Dubchak I. (2004). VISTA: Computational tools for comparative genomics. Nucleic Acids Res..

[37] Amiryousefi A., Hyvönen J., Poczai P. (2018). IRscope: An online program to visualize the junction sites of chloroplast genomes. Bioinformatics.

[38] Chen C., Chen H., Zhang Y., Thomas H.R., Frank M.H., He Y., Xia R. (2020). TBtools: An integrative toolkit developed for interactive analyses of big biological data. Mol. Plant.

[39] Rozas J., Ferrer-Mata A., Sanchez-DelBarrio J.C., Guirao-Rico S., Librado P., Ramos-Onsins S.E., Sanchez-Gracia A. (2017). DnaSP 6: DNA sequence polymorphism analysis of large data sets. Mol. Biol. Evol..

[40] Katoh K., Standley D.M. (2013). MAFFT multiple sequence alignment software version 7: Improvements in performance and usability. Mol. Biol. Evol..

[41] Darriba D., Taboada G.L., Doallo R., Posada D. (2012). JmodelTest 2: More models, new heuristics and parallel computing. Nat. Methods.

[42] Huelsenbeck J.P., Ronquist F. (2001). MRBAYES: Bayesian inference of phylogenetic trees. Bioinformatics.

[43] Saski C., Lee S.-B., Daniell H., Wood T.C., Tomkins J., Kim H.-G., Jansen R.K. (2005). Complete chloroplast genome sequence of *Glycine max* and comparative analyses with other legume genomes. Plant Mol. Biol..

[44] Cui Y., Nie L., Sun W., Xu Z., Wang Y., Yu J., Song J., Yao H. (2019). Comparative and phylogenetic analyses of ginger (*Zingiber officinale*) in the family Zingiberaceae based on the complete chloroplast genome. Plants.

[45] McInerney J. (1998). GCUA: General codon usage analysis. Bioinformatics.

[46] Zuo L.H., Shang A.Q., Zhang S., Yu X.Y., Ren Y.C., Yang M.S., Wang J.M. (2017). The first complete chloroplast genome sequences of *Ulmus* species by *de Novo* sequencing: Genome comparative and taxonomic position analysis. PLoS ONE.

[47] Suo Z., Jin X., Suo Z.L., Li W.Y., Jin X.B., Zhang H.J. (2016). A new nuclear DNA marker revealing both microsatellite variations and single nucleotide polymorphic loci: A case study on classification of cultivars in *Lagerstroemia indica*. Artic. J. Microb. Biochem. Technol..

[48] Zhang Y., Du L., Liu A., Chen J., Wu L., Hu W., Zhang W., Kim K., Lee S.C., Yang T.J. (2016). The complete chloroplast genome sequences of five *Epimedium* species: Lights into phylogenetic and taxonomic analyses. Front. Plant Sci..

[49] Chen J., Hao Z., Xu H., Yang L., Liu G., Sheng Y., Zheng C., Zheng W., Cheng T., Shi J. (2015). The complete chloroplast genome sequence of the relict woody plant *Metasequoia glyptostroboides* Hu et Cheng. Front. Plant Sci..

[50] Dong W.L., Wang R.N., Zhang N.Y., Fan W.B., Fang M.F., Li Z.H. (2018). Molecular evolution of chloroplast genomes of orchid species: Insights into phylogenetic relationship and adaptive evolution. Int. J. Mol. Sci..

[51] Park I., Yang S., Choi G., Kim W.J., Cheol M.B. (2017). The complete chloroplast genome sequences of *Aconitum pseudolaeve* and *Aconitum longecassidatum*, and development of molecular markers for distinguishing species in the *Aconitum* subgenus Lycoctonum. Molecules.

[52] Mehmood F., Abdullah, Shahzadi I., Ahmed I., Waheed M.T., Mirza B. (2020). Characterization of *Withania somnifera* chloroplast genome and its comparison with other selected species of Solanaceae. Genomics.

[53] Henriquez C.L., Abdullah, Ahmed I., Carlsen M.M., Zuluaga A., Croat T.B., McKain M.R. (2020). Evolutionary dynamics of chloroplast genomes in subfamily Aroideae (Araceae). Genomics.

[54] Wang R.J., Cheng C.L., Chang C.C., Wu C.L., Su T.M., Chaw S.M. (2008). Dynamics and evolution of the inverted repeat-large single copy junctions in the chloroplast genomes of monocots. BMC Evol. Biol..

[55] Huo Y., Gao L., Liu B., Yang Y., Kong S., Sun Y., Yang Y., Wu X. (2019). Complete chloroplast genome sequences of four *Allium* species: Comparative and phylogenetic analyses. Sci. Rep..

[56] Muraguri S., Xu W., Chapman M., Muchugi A., Oluwaniyi A., Oyebanji O., Liu A. (2020). Intraspecific variation within castor bean (*Ricinus communis* L.) based on chloroplast genomes. Ind. Crops Prod..

[57] Kim Y., Shin J., Oh D.R., Kim A.Y., Choi C. (2020). Comparative analysis of complete chloroplast genome sequences and insertion-deletion (Indel) polymorphisms to distinguish five *Vaccinium* species. Forests.

[58] Song W., Ji C., Chen Z., Cai H., Wu X., Shi C., Wang S. (2022). Comparative analysis the complete chloroplast genomes of nine *Musa* species: Genomic features, comparative analysis, and phylogenetic implications. Front. Plant Sci..

[59] Tian S., Lu P., Zhang Z., Wu J.Q., Zhang H., Shen H. (2021). Chloroplast genome sequence of chongming lima bean (*Phaseolus lunatus* L.) and comparative analyses with other legume chloroplast genomes. BMC Genomics.

[60] Alzahrani D.A., Yaradua S.S., Yaradua S.S., Albokhari E.J., Albokhari E.J., Abba A. (2020). Complete chloroplast genome sequence of *Barleria prionitis*, comparative chloroplast genomics and phylogenetic relationships among Acanthoideae. BMC Genomics.

[61] Wang X., Zhou T., Bai G., Zhao Y. (2018). Complete chloroplast genome sequence of Fagopyrum dibotrys: Genome features, comparative analysis and phylogenetic relationships. Sci. Rep..

[62] Yan C., Du J., Gao L., Li Y., Hou X. (2019). The complete chloroplast genome sequence of watercress (*Nasturtium officinale* R. Br.): Genome organization, adaptive evolution and phylogenetic relationships in Cardamineae. Gene.

[63] Li B., Zheng Y. (2018). Dynamic evolution and phylogenomic analysis of the chloroplast genome in Schisandraceae. Sci. Rep..

[64] Zong D., Gan P., Zhou A., Zhang Y., Zou X., Duan A., Song Y., He C. (2019). Plastome sequences help to resolve deep-level relationships of *Populus* in the family Salicaceae. Front. Plant Sci..

[65] Huang H., Shi C., Liu Y., Mao S.-Y., Gao L.Z. (2014). Thirteen Camellia chloroplast genome sequences determined by high-throughput sequencing: Genome structure and phylogenetic relationships. BMC Evol. Biol..

[66] Meng K.K., Chen S.F., Xu K.W., Zhou R.C., Li M.W., Dhamala M.K., Liao W.B., Fan Q. (2021). Phylogenomic analyses based on genome-skimming data reveal cyto-nuclear Discordance in the eolutionary history of Cotoneaster (Rosaceae). Mol. Phylogenet. Evol..

[67] Li P., Lu R.S., Xu W.Q., Ohi-Toma T., Cai M.Q., Qiu Y.X., Cameron K.M., Fu C.X. (2017). Comparative genomics and phylogenomics of East Asian tulips (*Amana*, Liliaceae). Front. Plant Sci..

[68] Nater A., Burri R., Kawakami T., Smeds L., Ellegren H. (2015). Resolving evolutionary relationships in closely related species with whole-genome sequencing data. Syst. Biol..

[69] Ma J., Clemants S. (2006). A history and overview of the Flora Reipublicae Popularis Sinicae (FRPS, Flora of China, Chinese Edition, 1959–2004). Taxon.

[70] Sang T. (2002). Utility of low-copy nuclear gene sequences in plant phylogenetics. Crit. Rev. Biochem. Mol..

[71] Li J., Christophel D.C., Conran J.G., Li H.W. (2004). Phylogenetic Relationships within the “core” *Laureae* (*Litsea* Complex, Lauraceae) Inferred from Sequences of the Chloroplast Gene *MatK* and Nuclear Ribosomal DNA *ITS* Regions. Plant Syst. Evol..

[72] Fijridiyanto I.A., Murakami N. (2009). Phylogeny of Litsea and Related Genera (Laureae-Lauraceae) based on analysis of *Rpb2* gene sequences. J. Plant Res..

[73] Srinivas S.G., Krishnamurthy Y.L., Kumar S.S. (2021). Diversity, ecology and molecular phylogeny of genus *Litsea* (Lauraceae) in central western ghat areas of India. Trop. Ecol..

[74] Li H.T., Yi T.S., Gao L.M., Ma P.F., Zhang T., Yang J.B., Gitzendanner M.A., Fritsch P.W., Cai J., Luo Y. (2019). Origin of angiosperms and the puzzle of the Jurassic Gap. Nat. Plants.

[75] Zhang Y., Tian Y., Tng D.Y.P., Zhou J., Zhang Y., Wang Z., Li P., Wang Z. (2021). Comparative chloroplast genomics of *Litsea* Lam. (Lauraceae) and its phylogenetic implications. Forests.

[76] Abou-Shaara H.F., Syed Abbas A., AL-Kahtani S.N., Taha E.K.A., Ali Khan K., Jamal Z.A., Alhumaidi Alotaibi M., Ahmad B., Ahmad Khan N., Qamer S. (2021). Exploring the Non-Coding regions in the MtDNA of some honey bee species and subspecies. Saudi J. Biol. Sci..

[77] Wu L.W., Cui Y.X., Wang Q., Xu Z.C., Wang Y., Lin Y.L., Song J.Y., Yao H. (2021). Identification and phylogenetic analysis of five *Crataegus* species (Rosaceae) based on complete chloroplast genomes. Planta.

[78] Liu Z.F., Ma H., Ci X.Q., Li L., Song Y., Liu B., Li H.W., Wang S.L., Qu X.J., Hu J.L. (2021). Can plastid genome sequencing be used for species identification in Lauraceae?. Bot. J. Linn. Soc..

[79] Chen Y.C., Li Z., Zhao Y.X., Gao M., Wang J.Y., Liu K.W., Wang X., Wu L.W., Jiao Y.L., Xu Z.L. (2020). The *Litsea* genome and the evolution of the laurel family. Nat. Commun..

