# Supplementary figures and images for: Comparative Analysis of Complete Chloroplast Genomes of Nine Species of Litsea (Lauraceae): Hypervariable Regions, Positive Selection, and Phylogenetic Relationships
Source: Genes (Basel). 2022 Aug 28;13(9):1550. doi: 10.3390/genes13091550 (PMC9498446; doi:10.3390/genes13091550)

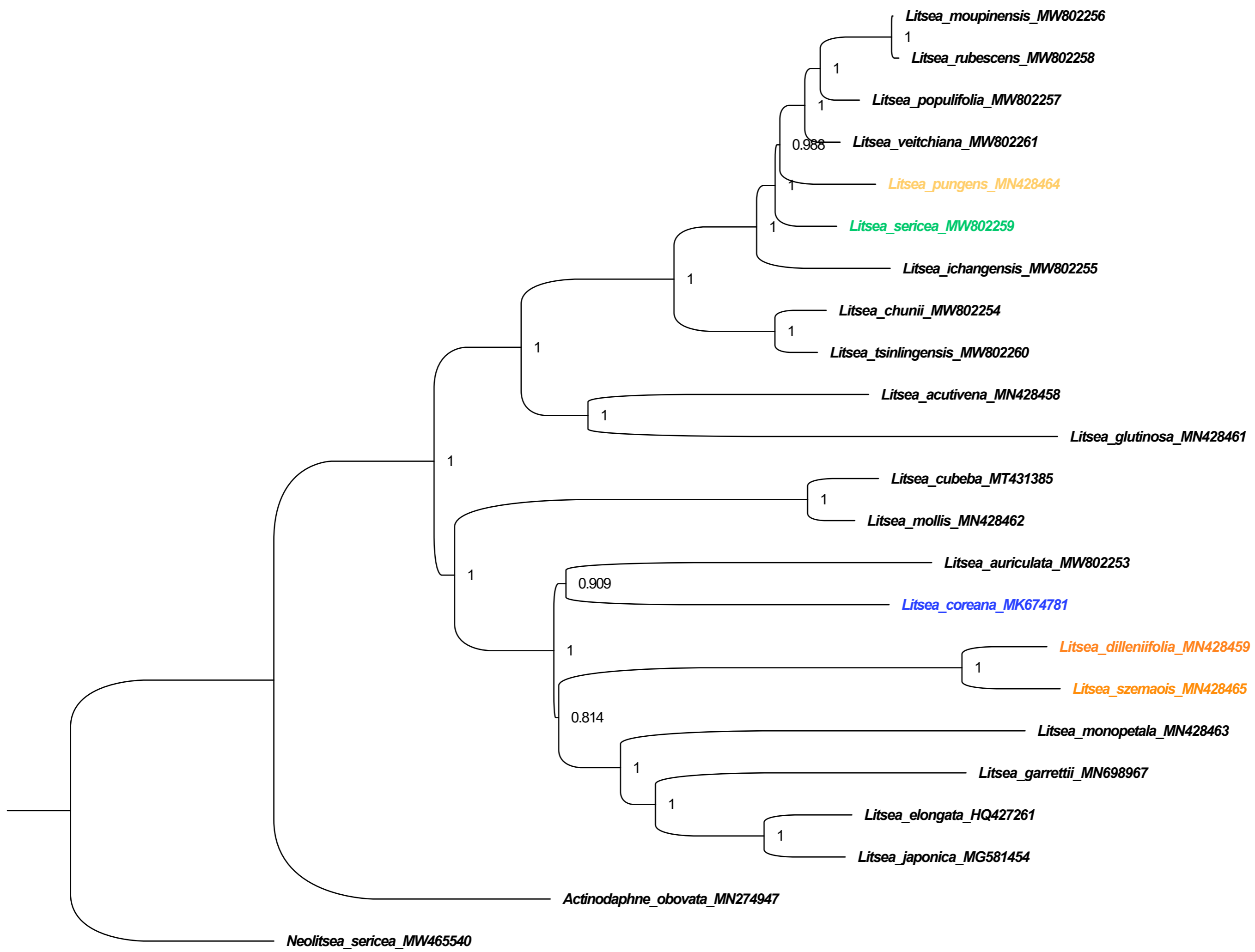

Supplement: Supplementary file 1 [file genes-13-01550-s001.zip › File S1.pdf]
